# Normative Data for Single- and Dual-Task Tandem Gait Performance in Collegiate Athletes

**DOI:** 10.1007/s40279-025-02306-2

**Published:** 2025-10-03

**Authors:** Eric J. Shumski, Landon B. Lempke, David Howell, Thomas Buckley, Jessie Oldham, William Meehan, Robert C. Lynall

**Affiliations:** 1 Concussion Research Laboratory, Department of Kinesiology, University of Georgia, Athens, GA USA; 2 Research in Athlete and Military Health Sciences Laboratory, Department of Physical Medicine and Rehabilitation, Virginia Commonwealth University School of Medicine, Richmond, VA USA; 3 Department of Orthopedics, Children’s Hospital Colorado and University of Colorado Anschutz Medical Campus, Aurora, CO USA; 4 Department of Kinesiology and Applied Physiology, University of Delaware, Newark, DE USA; 5 Division of Sports Medicine, The Micheli Center for Sports Injury Prevention, Waltham, MA USA; 6 Traumatic Brain Injury Center of Excellence, 5373 Gruber Road, Building C8837, Fort Bragg, NC USA

## Abstract

**Background:**

Normative dual-task (concurrent cognitive and motor task) tandem gait has not been developed. Currently, only individual baseline data are used for tandem gait assessment post concussion.

**Objective:**

The object was to (1) determine factors associated with single-task and dual-task tandem gait time among collegiate athletes across multiple institutions, and (2) provide robust normative data for single-task and dual-task tandem gait time based on clinically relevant factors.

**Methods:**

Data were analyzed from 2,137 unique collegiate athletes (19.0 ± 1.1 years, 48.9% female, 23.7% with concussion history) from 2015 to 2022 during pre-injury baseline concussion testing from three universities. Tandem gait was performed under single- and dual-task conditions (serial subtraction by sixes/sevens, spelling five-letter words backward, reciting the months backward). The criteria for being a clinically relevant independent variable was (a) *p* value < 0.05, and (b) effect estimate of ≥ 1 s. Normative data based on established percentile thresholds were derived and stratified by clinically relevant factors.

**Results:**

None of the single-task tandem gait times were clinically relevant, while sex and contact level were for dual task. Mean (95% confidence interval) for overall single- and dual-task tandem gait times were 12.07 s (11.95, 12.19) and 16.51 s (16.29, 16.73), respectively.

**Conclusion:**

Our results provide robust normative data for single- and dual-task tandem gait stratified by relevant patient factors that can be immediately used by clinicians and future researchers. Future research should compare the use of individual baseline versus normative data for acute concussion tracking.

**Supplementary Information:**

The online version contains supplementary material available at 10.1007/s40279-025-02306-2.

## Key Points

**Table:** 

Single-task tandem gait data are not influenced by demographic factors and should be interpreted as raw values.
Dual-task tandem gait is influence by sex and contact sport level and needs to be interpreted specifically with those variables. Normative data are provided and stratified by sex and contact level.

## Introduction

Concussions are often characterized by increased symptoms (e.g., headache, dizziness, difficulty concentrating), impaired cognitive capabilities, and/or impaired balance compared to baseline (pre-concussion) [1, 2]. The clinical balance assessment for concussion can be assessed through static (e.g., Balance Error Scoring System (BESS)) and dynamic (e.g., tandem gait) tasks. Both tests are included in the most recent international concussion consensus statement [1] and the Sport Concussion Assessment Tool 6 (SCAT6) [3]. Tandem gait requires a person/athlete to walk heel-to-toe, in a straight line, and over a pre-specified distance. Tandem gait is often paired with a concurrent cognitive condition to create a dual-task paradigm, which is a relatively newer evaluation in concussion literature and clinical practice compared to the BESS [1, 3]. Tandem gait displayed good-excellent inter-rater and test–retest reliability [4–6] while also having better discriminatory ability between concussed and control participants compared to BESS under single-task conditions (area under the concentration–time curve: 0.704 vs. 0.508) [7]. Dual-task tandem gait has not been directly compared to BESS in prior literature. However, dual-task tandem gait is consistently more challenging (i.e., longer time to completion) compared to single-task tandem [8–11], and is therefore likely to exhibit similar discriminatory properties. Tandem gait has better discriminatory ability than BESS because, potentially, it is a dynamic task requiring movement, incorporates a concurrent cognitive condition (i.e., dual-task), and has an objective scoring methodology (i.e., completion time via stopwatch), while the BESS is a static assessment, tested under single-task conditions, and is subjectively rated. We are focusing on dynamic balance normative data because symptoms, cognition, and the BESS already have established normative data [12–17], whereas tandem gait has limited data.

Preliminary normative data only exist for single-task tandem gait [18, 19], but these studies are not adequate for clinical use among collegiate athletes due to small samples size, the focus on adolescent populations, and insufficiently detailed normative data reporting. One study reported preliminary normative tandem gait among 94 professional hockey athletes who were 16–40 years old [19]. Another study reported tandem gait for 400 collegiate athletes [18], but only reported a group mean, 25th quartile, and 75th quartile single-task tandem gait values without consensus-based recommended normative ranges and without dual-task tandem gait times [20]. Due to both single- and dual-task conditions incorporated into the SCAT6 and SCOAT6 (either optional or recommended), more robust normative data containing all performance outcome metrics is warranted. Additionally, dual-task tandem gait assesses an athletes’ ability to multi-task (e.g., dual-task is the completion of a concurrent cognitive and motor task), which better emulates a sports competition environment.

The development of normative data is critical for effective clinical integration because baseline testing, although recommended or mandated, can be a time burden for athletes and clinicians with increased financial and personnel costs. Additionally, certain clinics outside of sports may not have any baseline data to rely on for comparisons. Balance may also be influenced by factors such as age, sex, or sport contact type [21–25], and therefore tailoring normative tandem gait data to influential factors can improve precision for clinicians determining if tandem gait performance is within an expected range. Although not all studies show demographic and sport type differences in tandem gait times [18, 26] robust normative data can thoroughly confirm or refute these findings. Further, in cases where pre-injury tandem gait baseline evaluations will be performed regardless, normative data still hold utility in identifying values outside of normal ranges and indicating potentially suboptimal athlete performance [27, 28].

Tandem gait is useful clinically because it does not require instrumented equipment, large amounts of space, and is not a significant time burden. Instrumented dual-task normal gait impairments may resolve at 2 months post concussion [29] but require expensive equipment and technological expertise to operate. Dual-task tandem gait resolves at the return to play clinical milestone post concussion [8] and only requires 3 m of space and a stopwatch (e.g., smartphone stopwatch). A single trial of tandem gait typically takes < 30 s to complete including processing time, whereas instrumented gait trials similarly take < 30 s to complete but the processing time will vary depending on the complexity of data. Therefore, tandem gait lowers the barrier to entry for dynamic balance assessments and can be readily used by clinicians without specialized training.

The purpose of the study was to (1) determine factors associated with single-task and dual-task tandem gait time among collegiate athletes across multiple institutions, and (2) provide robust normative data for single-task and dual-task tandem gait time based on the identified clinically relevant factors. Our results will provide clinicians with normative values across a large, heterogenous sample of athletes.

## Methods

### Equity, Diversity, and Inclusion Statement

To ensure our findings could be generalized as broadly as possible, we did not exclude any athletes from our study based on gender, sex, race, or ethnicity. Data collection was in-person and there may have been some language barriers as athletes come from all over the world to various colleges; however, physical demonstrations were provided when necessary and the data as a whole did not exclude athletes because of a language barrier, leading to more generalizable results. In the results and interpretation, we considered sex as a potential predictor variable due to prior research focusing on sex and tandem gait [18, 26].

### Inclusion/Exclusion Criteria and Consent

Data were prospectively collected and subsequently analyzed between the years 2015–2022 during pre-participation/pre-season baseline concussion testing from three Universities (Table 1). Athletes were included if they were NCAA athletes, cheerleaders, ≥ 18 years old, and provided written and informed consent. Athletes were excluded if they were currently recovering from a concussion (i.e., self-report if cleared to return to play by a medical provider) or lower extremity injury that prohibited them from performing tandem gait. All methods were approved by the Boston Children’s Hospital (P00016469), University of Georgia’s (STUDY00001479), and University of Delaware’s (804454) Institutional Review Boards. Patients and the public were not involved in the development or dissemination of this study. All procedures performed in our study were in accordance with the ethical standards of the institutional and/or national research committee and with the 1964 Helsinki Declaration and its later amendments or comparable ethical standards.

**Table 1:** Demographics

	*N* (%)	Mean (SD)	Median (IQR)
Institution			
University A	259 (12.1%)	–	–
University B	705 (33.0%)		
University C	1,173 (54.9%)	–	–
Age (years)	–	19.0 (1.1)	18.8 (18.0, 19.6)
Height (cm)	–	177.0 (11.4)	177.8 (167.6, 185.4)
Weight (kg)	–	77.0 (18.2)	75.0 (63.6, 86.4)
Concussion history frequency			
0	1602 (76.3%)	–	–
1	348 (16.6%)	–	–
2	117 (5.6%)	–	–
3 +	33 (1.6%)	–	–
Concussion history			
No	1602 (76.3%)	–	–
Yes	498 (23.7%)	–	–
SCAT total symptoms	–	2.3 (3.5)	1.0 (0.0, 3.0)
SCAT symptom severity	–	4.0 (7.9)	1.0 (0.0, 4.0)
Sex			
Female	1046 (48.9%)	–	–
Male	1091 (51.1%)	–	–
Sport classification			
Noncontact	489 (22.9%)	–	–
Limited	370 (17.3%)	–	–
Contact	1278 (59.8%)	–	–
Depression			
No	1371 (96.4%)	–	–
Yes	51 (3.6%)	–	–
Attention-deficit hyperactivity disorder			
No	1312 (92.3%)	–	–
Yes	110 (7.7%)	–	–
Learning disability^a^			
No	1725 (93.8%)	–	–
Yes	114 (6.2%)	–	–

Percentages may not add up to 100% due to rounding. *SCAT* Sport Concussion Assessment Tool

^a^Learning disability was anything that was reported as a learning disability but not attention-deficit hyperactivity disorder

### Collegiate-Athlete Baseline Demographics

Concussion history was self-reported with the National Institute of Neurological Disorders and Stroke and Department of Defense Sport-Related Concussion Common Data Elements forms and based on methods from the CARE consortium [30, 31]. Demographic information included: age, height, weight, sex (male/female), sport classification [32], as well as self-reported depression diagnosis (yes/no), ADHD diagnosis (yes/no), and learning disability diagnosis (yes/no). Sport classification was characterized as noncontact, limited, or contact based on the sport collegiate athletes participated in and the classification methods defined by Rice et al. [32].

The SCAT symptom checklist was used to collect total symptoms and symptom severity. Twenty-two symptoms were assessed on a scale of 0 (none) to 6 (severe). Outcomes were total symptom frequency and the sum of symptoms with a score ≥ 1 reported (range 0–22), and symptom severity was the sum of values (0–6) reported for each symptom (range 0–132) [33, 34]. All the demographic variables (e.g., sex, depression, symptoms) are our independent variables of interest in analyses.

### Single-Task Cognitive Measures

Prior to beginning tandem gait, cognitive measures of serial subtraction by sixes or sevens, spelling five-letter words backward, and reciting the months backward were completed at rest (i.e., not concurrently with tandem gait) to determine a cognitive condition baseline. Athletes completed at least one trial of each task and were instructed to perform the task as quickly and as accurately as possible for 20 s at University A. Universities B and C did not record their single-task cognitive measures. Athlete responses were tracked with pencil and paper in real time.

### Tandem Gait

Tandem gait was performed on a 3-m tape line while timed per instructions from the current and prior SCAT versions [3, 35, 36]. Athletes were required to walk heel-to-toe to the end of the tape line, turn around 180°, and walk heel-to-toe back to the starting position as quickly as possible (6 m total distance). A figure with the same instructions can be found in the article by Oldham et al. [18] and specific instructions can be found in the supplemental material of the SCAT6 [3]. Single-task tandem gait required athletes to perform only the motor task (i.e., tandem gait) as quickly as possible. Dual-task tandem gait required athletes to perform both the motor task and the cognitive task concurrently. Athletes were not instructed to prioritize one task over the other during the dual-task condition, and were told to perform both to the best of their abilities. No prioritization was given to mimic post-concussion assessment instructions and thereby allowing our normative data to be used by medical providers and clinicians who follow these same, standard of care/practice, instructions. The three cognitive tasks used during dual-task tandem gait were serial subtraction by sixes or sevens, spelling five-letter words backward, and reciting the months backward [10, 11]. A clinician or research team staff member selected the starting trial cognitive condition, or lack of cognitive condition (i.e., single-task), and ensured each cognitive condition was performed at least once. However, it was up to the clinician to choose their own order, thereby making the order unknown and completely random. These instructions were standardized across the three universities. At University A, five trials of each single- and dual-task condition were collected. At Universities B and C, four trials of each single- and dual-task condition were collected. All single-task and dual-task tandem gait trials were averaged respectively to their conditions. All five trials were averaged at University A, and all four trials were averaged at Universities B and C.

### Data Processing

Dual-task cost (%) was calculated using a previously established method (Eq. 1) [10, 37]. Negative dual-task cost values represent a decrease in performance (longer tandem gait time, slower response rate, worse percent correct, etc.) during the dual-task condition relative to the single-task condition. The sign of dual-task cost was flipped accordingly with the variable. For example, slower gait times during dual-task trials would equate to positive dual-task cost results, but we flipped the sign to negative because we felt negative dual-task cost was more intuitive for understanding worse performance during dual-task compared to single-task trials.

1 $${\text{Dual - Task}}\;{\mathrm{Cost}} = \frac{{\left( {{\text{dual - task}}\;{\mathrm{condition}}} \right) - \left( {{\text{single - task}}\;{\mathrm{condition}}} \right)}}{{\left( {{\text{single - task}}\;{\mathrm{condition}}} \right)}} \times 100.$$

Percent correct and correct response rate during the single-task cognitive measures and dual-task tandem gait performance were calculated for each condition separately (e.g., serial subtraction, spelling words backward), then averaged across all conditions (Eqs. 2 and 3). The calculation for single-task correct response rate was from the 20 s allotted for the cognitive tasks (see above Single-Task Cognitive Measures, Sect. 2.4). The calculation of the dual-task correct response rate used the tandem gait completion time. Athlete responses were tracked with pencil and paper in real-time.

2 $${\mathrm{Percent}}\;{\mathrm{Correct}} = \frac{{\left( {{\mathrm{Number}}\;{\mathrm{of}}\;{\mathrm{Correct}}\;{\mathrm{Responses}}} \right)}}{{\left( {{\mathrm{Number}}\;{\mathrm{of}}\;{\mathrm{Responses}}\;{\mathrm{Attempted}}} \right)}} \times 100$$

3 $${\mathrm{Correct}}\;{\mathrm{Response}}\;{\mathrm{Rate}}\;{\mathrm{per}}\;{\mathrm{Minute}} = \frac{{\left( {{\mathrm{Number}}\;{\mathrm{of}}\;{\mathrm{Correct}}\;{\mathrm{Responses}}} \right)}}{{\left( {{\mathrm{Tandem}}\;{\mathrm{Gait}}\;{\mathrm{Time}}} \right)}} \times 60.$$

### Statistical Analyses

For Aim 1, we used univariable general linear mixed models with restricted maximum likelihood estimation to determine which demographic factors were significantly associated with tandem gait outcomes. Institution was used as a random intercept in all models to account for any significant institutional-level variability to derive accurate associations between demographic factors and tandem gait outcomes (Online Supplementary Material Table 1).

Demographic factors were included for multivariable modeling and evaluated for interaction effects if they met clinically relevant criteria. The clinically relevant criteria for independent variables were (1) a *p* value < 0.05, and (2) effect estimate ≥ 1 s for an independent variable (e.g., sex (male/female), learning disability (yes/no)). We used a statistical and clinical threshold approach for modeling because the large sample size was likely to identify statistically significant findings regardless of the effect estimate magnitude. There is no existing literature for clinically relevant tandem gait differences between demographic variables to use as a cut-off. Additionally, there is no test–retest reliability for NCAA collegiate athletes (reliability only exists for recreationally active adults, adolescents, and older adults); therefore, using the minimal detectable change (MDC) or reliable change index as a cut-off was not possible. One study did report the MDC (~ 0.38 s), but the study only reported single-task tandem gait and had a small sample size [7]. The scenarios described above were discussed at length among the author team before the two rules (*p* < 0.05, effect estimate of ≥ 1 s) were created  a priori to analyses. The author team had two separate conference calls discussing the manuscript overall and the best approach for a clinically meaningful cut-off threshold. Consensus was reached on 1 s because there are no established reliable change indices, minimal detectable change values, or similar established cut points among any of our independent variables in collegiate samples. Some literature exists for biological sex and sport classification [18, 26], but they do not establish a cut-off. Additionally, four co-authors are athletic trainers and one co-author a physician who still works in a clinical environment, and all agree 1 s would offer balance in being sensitive to potentially clinically meaningful demographic effects while being conservative to statistically significant differences with low effect estimates attributed to the large sample size examined.

For the clinically relevant threshold, all were interpreted as a 1-unit increase or difference in the independent variables related to a ≥ 1-s increase in the outcome/dependent variable. For example, if a 1-unit change (e.g., from coded male to female) equated to a ≥ 1-s difference, then sex was considered clinically relevant. This was true for all independent variables except continuous variables (SCAT total symptoms, SCAT symptom severity, height, and weight). For SCAT total symptoms and SCAT symptom severity, we used an increase of four total symptoms and 7 symptom severity related to a ≥ 1-s increase in tandem gait time as derived from established 90% reliable change index values [38]. This means a 1 total symptom change equated to a 0.25-s change, and a 1 symptom severity change to a 0.14-s change. For height, we used a 15.24-cm (6-in.) threshold. For weight, we used a 4.54-kg. (10-lb) threshold. The tables below (Tables 3, 4) represent 1-s change in tandem gait time relating to a 1-cm and/or 1-kg change in height or weight. However, the interpretation for being included in the multivariable model is based on the description above about symptoms, height, and weight.

Demographic factors meeting the above clinically relevant thresholds were then included in a multivariable general linear mixed model with interaction terms. For example, if sex and concussion history met our clinically relevant criteria, then a sex × concussion history interaction term was evaluated for our multivariable models. We included Institution as a random intercept in the multivariable model. The demographic variables in the final multivariable model were considered significant based on *p* values (< 0.05) alone. The lack of clinical cut-off values in the multivariable analyses was considered unnecessary because we already considered the clinical cut-off in the univariable analyses. Bonferroni adjustments were made for univariable and multivariable analyses when necessary (three-level adjustment *α* = 0.017; six-level adjustment *α* = 0.008).

For Aim 2, we provided normative data for single- and dual-task tandem gait times by each significant factor from the multivariable model in Aim 1. Normative tandem gait data were characterized by consensus reporting guidelines from the American Academy of Clinical Neuropsychology categories [20]. These data were normally distributed, so the following categories were used: exceptionally high score (percentile: ≥ 98), above average score (percentile: 91–97), high average score (percentile: 75–90), average score (percentile: 25–74), low average score (percentile: 9–24), below average score (percentile: 2–8), exceptionally low score (percentile: < 2) [20]. We did not create normative values based on clinically relevant factors for dual-task cost, percent correct, or response rate as they are rarely used in clinical practice and not scored as outcomes on the SCAT6 or SCOAT6. However, we did create normative values for the raw data (e.g., not split by clinically relevant factors) for future research use (Online Supplementary Material Tables 2–8).

## Results

Participant demographics from 2137 collegiate athletes are reported in Table 1, and are similar to large datasets of NCAA athletes [2, 39, 40]. The cumulative cohort was most commonly from University C (54.9%), played contact sports (59.8%), had no prior concussion history (76.2%), and approximated equal sex representation (48.9% female; Table 1). Descriptive statistics for cognitive measures and dual-task cost measures are reported in Table 2.

**Table 2:** Cognitive and dual-task cost outcomes

	*N* (%)	Mean (SD)	Median (IQR)
Single-task percent correct (%)^a^	259 (12.1%)	91.5 (9.8)	94.4 (88.6, 98.7)
Single-task response rate (#/min)^a^	259 (12.1%)	23.7 (7.6)	23.0 (18.3, 28.3)
Dual-task percent correct (%)	1,769 (82.3%)	83.8 (20.8)	90.6 (77.5, 100.0)
Dual-task response rate (#/min)	1427 (66.8%)	21.5 (9.2)	21.5 (16.2, 27.5)
Dual-task cost percent correct^a^ (%)	258 (12.1%)	− 4.2 (12.3)	− 3.1 (− 9.5, 1.9)
Dual-task cost response rate^a^ (#/min)	258 (12.1%)	− 2.5 (21.3)	− 3.2 (− 13.6, 9.9)
Dual-task cost tandem gait time (%)	1949 (91.2%)	− 37.8 (33.0)	− 31.4 (− 50.8, 15.3)

Negative dual-task cost values represent a decrease in performance (e.g., longer tandem gait times, worse cognitive performance) during the dual-task condition compared to the single-task condition

^a^Data are from University A only. Universities B and C did not collect single-task cognitive measures

### Aim 1

#### Single-Task Tandem Gait Time

Males had significantly faster single-task tandem gait time than females (*p* < 0.001). The taller (*p* < 0.001) and heavier (*p* < 0.001) an athlete was, the faster they completed the single-task tandem gait task. However, none of the factors met our clinically relevant threshold of ≥ 1 s (Table 3). Therefore, no multivariable single-task tandem gait model was conducted.

**Table 3:** Univariable associations with single-task tandem gait time

	Random effects		Fixed effects	
	Institution variance	Residual variance	Estimate (s) (95% CI)	*p* value
Sex*	2.21	6.03	− 0.74 (− 0.96, − 0.52)	< 0.001
Sport classification				
Contact-limited contact	2.14	6.16	− 0.12 (− 0.48, 0.25)	0.999
Contact-noncontact			0.05 (− 0.28, 0.38)	0.999
Limited contact-noncontact			0.17 (− 0.26, 0.59)	0.999
Concussion history (yes/no)	2.13	6.15	0.12 (− 0.15, 0.38)	0.385
Depression (yes/no)	4.28	4.80	0.20 (− 0.41, 0.82)	0.515
ADHD (yes/no)	4.30	4.80	0.07 (− 0.35, 0.50)	0.748
Learning disability (yes/no)	2.19	5.83	− 0.17 (− 0.63, 0.30)	0.482
Age (years)	2.10	6.14	− 0.14 (− 0.23, − 0.05)	0.003
Height (cm)*	2.26	6.07	− 0.02 (− 0.03, − 0.01)	< 0.001
Weight (kg)*	2.22	6.12	− 0.01 (− 0.01, − 0.01)	< 0.001
Total no. of prior concussions	2.13	6.15	0.07 (− 0.10, 0.23)	0.430
SCAT total symptoms	2.14	5.96	0.01 (− 0.02, 0.05)	0.559
SCAT symptom severity	2.15	5.96	< 0.01 (− 0.01, 0.02)	0.620

Random effect was Institution. Learning disability was anything not including ADHD. Sex is reported as male relative to female. Sport classification is reported as contact relative to limited contact, contact relative to noncontact, and limited contact relative to noncontact. Concussion history, depression, ADHD, and learning disability are reported as yes relative to no

*ADHD* attention-deficit hyperactivity disorder, *SCAT* Sport Concussion Assessment Tool

*p < 0.05

#### Dual-Task Tandem Gait Time

Male athletes had a significantly faster dual-task tandem gait time compared to female athletes (*p* < 0.001). The overall model for sport classification was significant (*p* < 0.001), and after Bonferroni adjustments, contact athletes displayed a significantly faster dual-task tandem gait time (*p* < 0.001) than noncontact athletes. Additionally, as athletes got older (*p* < 0.001), taller (*p* < 0.001), and heavier (*p* < 0.001) they displayed faster dual-task tandem gait times. Though statistically significant, only sex and sport classification met our clinically relevant threshold of ≥ 1 s (Table 4).

**Table 4:** Univariable associations with dual-task tandem gait time

	Random effects		Fixed effects	
	Institution variance	Residual variance	Estimate (s) (95% CI)	*p* value
Sex*^a^	7.01	21.25	− 1.55 (− 1.97, − 1.14)	< 0.001
Sport classfication^a^				
Contact-limited contact	6.73	21.61	− 0.55 (− 1.23, 0.139)	0.169
Contact-noncontact*			− 1.21 (− 1.83, − 0.58)	< 0.001
Limited contact-noncontact			− 0.66 (− 1.46, 0.13)	0.138
Concussion history (yes/no)	7.04	22.00	− 0.25 (− 0.75, 0.25)	0.322
Depression (yes/no)	12.45	21.89	0.25 (− 1.05, 1.56)	0.705
ADHD (yes/no)	12.48	21.89	0.13 (− 0.78, 1.04)	0.779
Learning disability (yes/no)	6.87	21.97	− 0.22 (− 1.13, 0.69)	0.642
Age (years)*	6.75	21.62	− 0.41 (− 0.58, − 0.23)	< 0.001
Height (cm)*	7.43	21.51	− 0.04 (− 0.06, − 0.02)	< 0.001
Weight (kg)*	7.32	21.36	− 0.03 (− 0.04, − 0.02)	< 0.001
Total no. of concussions	7.08	22.00	− 0.16 (− 0.47, 0.15)	0.306
SCAT total symptoms	7.08	22.41	− 0.01 (− 0.07, 0.06)	0.881
SCAT symptom severity	7.08	22.41	< − 0.01 (− 0.03, 0.03)	0.869

Random effects were Institution. Learning disability was anything not including ADHD. Sex is reported as male relative to female. Sport classification is reported as contact relative to limited contact, contact relative to noncontact, and limited contact relative to noncontact. Concussion history, depression, ADHD, and learning disability are reported as yes relative to no

*ADHD* attention-deficit hyperactivity disorder, *SCAT* Sport Concussion Assessment Tool

*p < 0.05

^a^Meets clinically relevant threshold for inclusion in multivariable analyses

#### Dual-Task Tandem Gait Multivariable Model

Sex and sport classification met our clinically relevant criteria (Table 4). Our multivariable analysis with Institution as a random effect revealed no significant sex by sport classification interaction (*F* = 2.37, *p* = 0.093), but significant sex (*F* = 15.40, *p* < 0.001) and sport classification (*F* = 6.77, *p* = 0.001) main effects. The variance due to Institution and the residuals were 7.03 and 21.14, respectively. Female athletes walked significantly slower than male athletes (mean difference (95% CI) 1.61 s (1.12, 2.10), *p* < 0.001). After Bonferroni adjustments, there were no significant differences between contact, limited contact, or noncontact sports (*p*-range = 0.063–0.999).

### Aim 2

#### Normative Single-Task Tandem Gait Time

None of the demographic factors had an estimate above our clinically relevant criteria. Therefore, single-task tandem gait times were provided collectively for the entire sample (Table 5). The overall mean (95% CI) and median (IQR) were 12.07 (11.95, 12.19) s and 11.79 (10.44, 13.19) s, respectively.

**Table 5:** Normative single-task tandem gait time classification

	Exceptionally high score	Above average score	High above average score	Average	Lower average score	Below average score	Exceptionally low score
Percentile	≥ 98	97–91	90–75	74–25	24–9	8–2	< 2
Single-task tandem gait time (s)	4.57 − 7.11	7.12 − 8.85	8.86 − 10.43	10.44 − 13.18	13.19 − 15.85	15.86 − 19.48	19.49 − 27.61

*n* = 1951

#### Normative Dual-Task Tandem Gait Time

The normative data for dual-task tandem gait is tabulated by sex and sport classification in Table 6. The overall mean (95% CI) and median (IQR) were 16.51 (16.29, 16.73) s and 15.65 (13.01, 19.04) s, respectively. Multicollinearity was not present in the model. When ran as a linear model the variance inflation factor was 1.76–3.50, and the Spearman correlation between sex and sport classification was rho = 0.249 [41, 42].

**Table 6:** Normative dual-task tandem gait times

	Exceptionally high score	Above average score	High above average score	Average	Lower average score	Below average score	Exceptionally low score
Percentile	≥ 98	97–91	90–75	74–25	24–9	8–2	< 2
Male (*n* = 976)							
Noncontact (*n* = 131)	7.17–8.29	8.30–10.81	10.82–12.98	12.99–19.72	19.73–22.58	22.59–29.92	29.93–35.06
Limited contact (*n* = 151)	5.77–8.55	8.56–11.06	11.07–13.04	13.05–18.18	18.19–22.75	22.76–26.71	26.72–29.31
Contact (*n* = 694)	6.42–8.49	8.50–10.76	10.77–12.58	12.59–17.89	17.90–21.72	21.73–25.93	25.94–31.76
Female (*n* = 973)							
Noncontact (*n* = 313)	6.82–9.43	9.44–11.25	11.26–13.50	13.51–20.76	20.77–26.98	26.97–32.00	32.01–41.95
Limited contact (*n* = 201)	8.84–9.98	11.68–9.99	11.69–13.27	13.28–21.05	21.06–25.81	25.82–32.22	32.23–41.36
Contact (*n* = 459)	7.86–9.28	9.29–11.52	11.53–13.35	13.36–18.90	18.91–23.19	23.20–32.03	32.04–38.42

## Discussion

Our findings provide robust normative data for single- and dual-task tandem gait derived from a large, multi-institution dataset. While prior literature provided some single-task normative tandem gait data [18, 19], our study provides both single- and dual-task data, with usable normative threshold values, and are stratified by data-driven covariates. For instance, single-task gait time had no clinically relevant covariates (e.g., age, sex, height). Dual-task tandem gait time models included sex and sport classification as covariates, which should be considered by clinicians when reading Table 6 in the Results, Sect. 3.

Prior literature has focused on single-task tandem gait normative data among collegiate athletes (*n* = 400) [18] and male-only, professional hockey players (*n* = 304) [19]. On average from prior literature, the single-task tandem gait time for collegiate athletes and professional hockey players was 11.32 s and 10.80 s, respectively [19]. Our larger sample across most NCAA sports shows the average single-task tandem gait time to be 11.79 s (25th–74th percentile = 10.44–13.18 s) (Table 5), which lines up with previous literature. However, prior research did not observe concussion history, sport type, or sex to significantly affect single-task tandem gait time [18]. Our results revealed that single-task tandem gait was significantly different between males and females, but did not reach our clinically relevant threshold to be included in the multivariable analysis (Table 4). Our significant results compared to the previous literature’s [18] non-significant results are likely due to the larger sample size and inclusion of other Institutions.

There are no dual-task tandem gait normative data sets in the existing literature. Smaller sample size studies among collegiate athletes (*n* = 126) have shown no differences in dual-task tandem gait time between athletes with (18.82 s) and without (20.52 s) a concussion history [10]. Additionally, collegiate athletes (*n* = 114) with < 7 h of sleep displayed an average of 12.30 s, while athletes with ≥ 7 h of sleep displayed an average of 14.50 s on dual-task tandem gait times [43]. To our knowledge, no other literature has evaluated dual-task tandem gait times that were not acutely post-concussion. Our sample in the current study displayed an average time of 16.51 s, right in between the two prior studies. However, neither previous study investigated whether sex or sport classification was associated with dual-task tandem gait time, whereas our study showed both factors to be clinically relevant. Collegiate athletes when performing normal walking exhibit differences in spatiotemporal gait metrics (e.g., step length, step width, double support time/phase) between sex and sport classification (noncontact, limited, contact) [44]. Further, male high school athletes (*n* = 83) perform dual-task tandem gait faster than female high school athletes (*n* = 117) [45]. Our study provides normative dual-task tandem gait performance (Table 6) for collegiate athletes to be used by clinicians, and information regarding the significance of sex and sport classification at both univariable and multivariable levels for future research to consider.

Sport classification was clinically significant in our study, which contradicts some existing literature. A systematic review reported limited effects on static and dynamic postural control among populations with repetitive head impacts [46]. Normal dual-task gait is also unaffected by sport classification in collegiate athletes [47] and early-middle adulthood [48]. For tandem gait specifically, single-task tandem gait is unaffected by sport classification [18], but dual-task tandem gait has not been explored in relation to sport classification until our study (at least not with a large, representative sample size). The combination of tandem gait and a cognitive component likely led to the results found in our sample. Tandem gait requires greater balance and postural control compared to normal gait due to the smaller base of support, and coupled with a concurrent cognitive task leads to an even greater challenge that appeared to illicit differences at the opposite ends of sport classification in our sample (contact vs. noncontact).

Depression, ADHD, and learning disabilities have been shown to influence normal spatiotemporal gait characteristics [49–52], but ours is the first study to examine their association with tandem gait performance. We found no statistical differences in single- or dual-task tandem gait performance among these characteristics (Table 3). This is somewhat surprising based on prior literature, but our sample is made of collegiate athletes who are near the top of their performance capabilities and self-reported medical history. It is also possible that medication use among collegiate athletes mitigated any negative performance obstacles due to depression, ADHD, or learning disabilities. However, this is speculation as we did not track medication use. It may also be as simple as the fact that these learning and developmental disorders do not impact tandem gait in a similar manner to normal gait.

Height and weight were significantly related to single- and dual-task tandem gait performance; however, both were clinically irrelevant (i.e., very small estimates) (Table 3). Anecdotally, height and foot size are often considered to be important when considering tandem gait as height associates with foot size, and therefore taller individuals cover the 6-m distance in fewer steps (i.e., fleading to a faster time). A study of high school students showed height is related to tandem gait performance [45]. However, our large sample study showed height was not a clinically meaningful contributor for collegiate athletes. Weight was also statistically significant, but highly likely a redundant artifact due to height and weight being strongly correlated in our sample (*r* = 0.760, *p* < 0.001).

Although we statistically controlled for differences between Institutions (Cohen’s d range = 0.21–1.15; Online Supplementary Material Table 1) to derive more precise normative data, it is still an important factor to address. The tandem gait protocols and instructions for performance were the same between Institutions, but we still observed institutional-based differences. Potential reasons for differences between universities are unknown and could be due to random chance or a greater Hawthorne effect at one university versus another. These unknown differences highlight the relevance of controlling for institutional-level differences, and why we did, which ultimately improves the external validity and estimate precision of our normative data for clinical concussion diagnostics in various athletic environments. In other words, any unknown causes for the differences between universities are likely uncontrollable and reflect what medical providers and clinicians will experience in the real-world, thereby making our results more externally valid.

### Clinical Implications

Our robust single- and dual-task tandem gait normative data set can be immediately used by clinicians to determine if an athlete is performing above, below, or at average. The normative data can also be used to compare acute post-concussion tandem gait performance to inform whether impairments are likely present in the absence of pre-injury baseline data. On a large scale, our results will aid in the future determination of the necessity of individual baseline data for tandem gait, which is important to ascertain as baselining requires a large amount of people-power, time, and resources. Although baseline testing is currently mandated for collegiate athletics [53, 54], future investigations should compare the diagnostic accuracy of tandem gait between normative and individual baseline data. Evidence from other concussion assessments shows normative data performs equally as well as individual baseline data [14, 55, 56]. Even if diagnostic accuracy is equivalent between normative and individual baselines, a multimodal assessment post-concussion is still recommended due to multiple different symptoms and consequences a concussion can incur [1].

Although future research needs to determine the utility of normative data versus individual baseline data, there are some explicit uses clinicians can take advantage of with our current data. For example, clinicians can recognize that athletes in the “exceptionally low score” category have 98% of all athletes performing faster times. Clinicians can then make their own judgements about whether this category is appropriate or if there may be other reasons for the low score such as poor motivation. We do not have a recommendation for assessing these other reasons and leave it up to clinical judgement on a case-by-case basis.

### Limitations

Our study only examined NCAA Division I college athletes and may limit generalizability to other Divisions or outside the USA. Second, the majority of our demographic variables were self-reported. While self-reporting is common practice in research and medical preparticipation examinations, it may slightly dampen the validity of our results. Third, only one university collected single-task cognitive data, which limits its use clinically and overall generalizability as the results are biased to that single university. Lastly, the authors deliberated extensively to determine the best way to create clinically relevant threshold values for inclusion in the final, normative data tables. Although it was rigorously discussed, there was no clear delineation of importance for what is considered clinically relevant in prior literature or generally accepted among working clinicians. Based on the existing (or lack of) literature, we are confident in our 1-s cut-off as our author team consists of tandem gait and concussion experts, certified athletic trainers, and a physician. A major strength of our study was the near-equal representation of males and females as females are chronically under-represented in sports medicine [57] and concussion research [58].

## Conclusion

We found no independent variables associated with single-task tandem gait, while sex and sport classification were significantly associated with dual-task tandem gait. Our results provide robust normative data for single- and dual-task tandem gait stratified by relevant athlete characteristics that can be immediately used by clinicians and future researchers for concussion diagnostics in the absence of pre-injury tandem gait data.

## Supplementary Information

Below is the link to the electronic supplementary material.

Supplementary file1 (DOCX 37 kb)

## Declarations

### Code availability

The R code used for analysis is available upon reasonable request to the first author.

### Data Availability

Data are available upon reasonable request to the first author.
