# Supplementary material for: Normative Data for Single- and Dual-Task Tandem Gait Performance in Collegiate Athletes
Source: Sports Med. 2025 Oct 3;56(2):559–71. doi: 10.1007/s40279-025-02306-2 (PMC12982242; doi:10.1007/s40279-025-02306-2)
Supplement: Supplementary file 1 — Supplementary file1 (DOCX 37 kb) [file 40279_2025_2306_MOESM1_ESM.docx]

**Authors:** Eric J. Shumski PhD^1,*^, Landon B. Lempke, PhD, ATC^2^, David Howell, PhD, ATC^3^, Thomas Buckley EdD, ATC^4^, Jessie Oldham, PhD^2^, William Meehan MD^5^, Robert C. Lynall, PhD, ATC^1^

institution name, department, city [state], country

1. University of Georgia, Concussion Research Laboratory, Department of Kinesiology, Athens, Georgia, United States
2. Virginia Commonwealth University School of Medicine, Research in Athlete and Military Health Sciences Laboratory, Department of Physical Medicine and Rehabilitation, Richmond, Virginia, United States
3. Children’s Hospital Colorado and University of Colorado Anschutz Medical Campus, Department of Orthopedics, Aurora, Colorado, United States
4. University of Delaware, Department of Kinesiology and Applied Physiology, Newark, Delaware, United States
5. The Micheli Center for Sports Injury Prevention, Division of Sports Medicine, Waltham, Massachusetts, United States

* At the time of publishing, the corresponding author (EJS) is working as a contractor at General Dynamics Information Technology in support of the Traumatic Brain Injury Center of Excellence

**Corresponding Author:**

Eric Shumski, PhD

Traumatic Brain Injury Center of Excellence,

5373 Gruber Road, Building C8837, Fort Liberty, North Carolina

Phone: 910.908.2282

Email: ericshumski@gmail.com

| **Supplementary Table 1. Tandem Gait times by Institution (mean (95% confidence interval))** | | | |
| --- | --- | --- | --- |
|  | **University A** | **University B** | **University C** |
| Single-Task Tandem Gait Time (s) | 14.17  (13.80, 14.54) | 12.90  (12.63, 13.12) | 11.24  (11.12, 11.35) |
| Dual-Task Tandem Gait Time (s) | 20.60  (19.92, 21.29) | 16.54  (16.14, 16.95) | 15.59  (15.34, 15.85) |
| **P-values and Cohen’s d for Tandem Gait** | | | |
|  | **University A-University B**  **(p-value)**  **(Cohen’s d)** | **University B-**  **University C**  **(p-value)**  **(Cohen’s d)** | **University A-**  **University C**  **(p-value)**  **(Cohen’s d)** |
| Single-Task Tandem Gait Time (s) | p<0.001  d=0.41 | p<0.001  d=0.63 | p<0.001  d=1.15 |
| Dual-Task Tandem Gait Time (s) | p<0.001  d=0.79 | p=0.004  d=0.21 | p<0.001  d=0.99 |

| **Supplementary Table 2. Normative Single-Task Percent Correct Classification** | | | | | | | |
| --- | --- | --- | --- | --- | --- | --- | --- |
|  | Exceptionally High Score | Above Average score | High Above Average Score | Average | Lower Average Score | Below Average Score | Exceptionally Low Score |
| Percentile | ≥98 | 97-91 | 90-75 | 74-25 | 24-9 | 8-2 | <2 |
| Percent Correct (%) | 100.0-100.0 | 100.0-100.0 | 99.9-98.7 | 98.6-88.4 | 88.3-77.8 | 77.7-56.9 | 56.8-47.2 |
| n=259 | | | | | | | |

| **Supplementary Table 3. Normative Single-Task Correct Response Rate Classification** | | | | | | | |
| --- | --- | --- | --- | --- | --- | --- | --- |
|  | Exceptionally High Score | Above Average score | High Above Average Score | Average | Lower Average Score | Below Average Score | Exceptionally Low Score |
| Percentile | ≥98 | 97-91 | 90-75 | 74-25 | 24-9 | 8-2 | <2 |
| Correct Response Rate (#/min) | 45.0-41.1 | 41.0-34.5 | 34.4-28.0 | 27.9-18.0 | 17.9-14.5 | 14.4-8.1 | 8.0-4.5 |
| n=259 | | | | | | | |

| **Supplementary Table 4. Normative Dual-Task Percent Correct Classification** | | | | | | | |
| --- | --- | --- | --- | --- | --- | --- | --- |
|  | Exceptionally High Score | Above Average score | High Above Average Score | Average | Lower Average Score | Below Average Score | Exceptionally Low Score |
| Percentile | ≥98 | 97-91 | 90-75 | 74-25 | 24-9 | 8-2 | <2 |
| Percent Correct (%) | 100.0-100.0 | 100.0-100.0 | 100.0-100.0 | 99.9-77.6 | 77.5-58.4 | 58.3-5.6 | 5.5-0.0 |
| n=1769 | | | | | | | |

| **Supplementary Table 5. Normative Dual-Task Correct Response Rate Classification** | | | | | | | |
| --- | --- | --- | --- | --- | --- | --- | --- |
|  | Exceptionally High Score | Above Average score | High Above Average Score | Average | Lower Average Score | Below Average Score | Exceptionally Low Score |
| Percentile | ≥98 | 97-91 | 90-75 | 74-25 | 24-9 | 8-2 | <2 |
| Correct Response Rate (#/min) | 58.7-39.6 | 39.5-34.0 | 33.9-27.5 | 27.4-16.2 | 16.1-8.6 | 8.5-0.1 | 0.0-0.0 |
| n=1427 | | | | | | | |

| **Supplementary Table 6. Normative Dual-Task Cost Percent Correct Classification** | | | | | | | |
| --- | --- | --- | --- | --- | --- | --- | --- |
|  | Exceptionally High Score | Above Average score | High Above Average Score | Average | Lower Average Score | Below Average Score | Exceptionally Low Score |
| Percentile | ≥98 | 97-91 | 90-75 | 74-25 | 24-9 | 8-2 | <2 |
| Dual-Task Cost Percent Correct (%) | 60.2-22.7 | 22.6-7.2 | 7.1-1.9 | 1.8- -9.6 | -9.7- -16.9 | -17.0- -39.8 | -39.9- -53.3 |
| n=258 | | | | | | | |

| **Supplementary Table 7. Normative Dual-Task Cost Correct Response Rate Classification** | | | | | | | |
| --- | --- | --- | --- | --- | --- | --- | --- |
|  | Exceptionally High Score | Above Average score | High Above Average Score | Average | Lower Average Score | Below Average Score | Exceptionally Low Score |
| Percentile | ≥98 | 97-91 | 90-75 | 74-25 | 24-9 | 8-2 | <2 |
| Dual-Task Cost Correct Response Rate (%) | 124.8-36.1 | 36.0-23.4 | 23.3-9.9 | 9.8- -13.6 | -13.7- -24.4 | -24.5- -59.9 | -60.0- --65.8 |
| n=258 | | | | | | | |

| **Supplementary Table 8. Normative Dual-Task Cost Tandem Gait Times Classification** | | | | | | | |
| --- | --- | --- | --- | --- | --- | --- | --- |
|  | Exceptionally High Score | Above Average score | High Above Average Score | Average | Lower Average Score | Below Average Score | Exceptionally Low Score |
| Percentile | ≥98 | 97-91 | 90-75 | 74-25 | 24-9 | 8-2 | <2 |
| Dual-Task Cost Tandem Gait Times (%) | 275.3-127.7 | 127.6-83.5 | 83.4-50.8 | 50.7-15.3 | 15.2-4.3 | 4.2- -5.3 | -5.4- -30.5 |
| n=1949 | | | | | | | |
